# The effects of nano‐zinc oxide morphology on functional and antibacterial properties of tapioca starch bionanocomposite

**DOI:** 10.1002/fsn3.2426

**Published:** 2021-06-21

**Authors:** Naser Tamimi, Abdorreza Mohammadi Nafchi, Hamid Hashemi‐Moghaddam, Homa Baghaie

**Affiliations:** ^1^ Chemical Engineering Department Damghan Branch Islamic Azad University Damghan Iran; ^2^ Food Science and Technology Department Damghan Branch Islamic Azad University Damghan Iran; ^3^ Food Technology Division School of Industrial Technology Universiti Sains Malaysia USM Penang Malaysia

**Keywords:** antibacterial activity, morphology, nanotechnology, tapioca starch

## Abstract

The purpose of this study was to evaluate the effect of nano‐zinc oxide (ZnO‐N) morphology on the functional and antimicrobial properties of tapioca starch films. For this reason, nanosphere (ZnO‐ns), nanorod (ZnO‐nr), and nanoparticle of ZnO (ZnO‐np) at 0.5%, 1.0%, and 2.0% were added to the starch film. Then, physicochemical, mechanical, and barrier properties were evaluated. Also, UV–visible and Fourier transform infrared spectroscopy (FTIR) spectra and antibacterial activity of prepared nanocomposite films against *Escherichia coli* were examined. The results revealed that the ZnO‐ns had the most effects on mechanical, physicochemical, and barrier properties. The highest values of the tensile strength (14.15 MPa) and Young's modulus (32.74 MPa) and the lowest values of elongation at break (10.40%) were obtained in the films containing 2% of ZnO nanosphere. In terms of UV transmission, ZnO‐nr showed the most significant impact morphology. FTIR spectra indicated that interactions for all morphologies were physical interaction, and there are no chemical reactions between starch structure and nanoparticles. The antibacterial effect of the ZnO‐ns was higher than that of other morphologies. In summary, ZnO‐ns was the best morphology for using ZnO‐N in starch‐based nanocomposite films.

## Graphical abstract

Morphology of the nanoparticles is a key parameter in bionanocomposite characteristics. Spherical shape of the nanoparticles is the best shape for antibacterial, physicochemical, and mechanical properties of bionanocomposites. Rod shape of the nanoparticles is the best shape for UV shielding of bionanocomposite packaging.

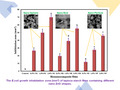

## 1 INTRODUCTION

Petroleum polymers have significant thermal, inhibitory, and mechanical properties and relatively low price and easy processing capability. Therefore, these polymers are widely used in the packaging industry (Abdullah & Dong, 2019; Araghi et al., 2015; Moslehi et al., 2021; Mousavian et al., 2021). However, plastic waste is on the rise and is likely to reach more than 12,000 million metric tons by 2050 (Geyer et al., 2017). Therefore, the food packaging industry is trying to reduce the consumption of plastic polymers and replace them with renewable and natural biopolymers obtained from different sources (Wang et al., 2020; Zemljič et al., 2020). Various biopolymers such as lipids, polysaccharides, and proteins have been used for packaging purposes (Ekramian et al., 2021). Starches are among the most widely used biopolymers in food packaging (Arezoo et al., 2020; Ekramian et al., 2021).

Starch is a unique carbohydrate and consists of individual granules. These granules are insoluble in water and are partially hydrated in cold water. Therefore, starches form solutions with low viscosity and are easily mixed or pumped. Starch is a renewable resource and has the ability to making films. Since starch has high availability and good extraction yield and is biodegradable, it can be used as a natural biopolymer to prepare edible films or coatings (Kowalczyk & Baraniak, 2014). Tapioca starch (*Manihot Esculenta Crantz*.) as a gelling and thickening agent and an adhesive for paper is used in food and nonfood industries. Compared to other starch sources, its price in the world market is low (Chaisawang & Suphantharika, 2006). Tapioca starch films have unique characteristics such as colorless, odorless, tasteless, nontoxic, and semipermeability to oxygen, CO_2_, lipids, flavor compounds, and moisture (Shah et al., 2015). This starch is also edible and suitable for food packaging systems and can minimize waste (Othman et al., 2019). However, starch films have low thermal and mechanical properties, and by using different methods, the weakness of these films must be overcome (Jayakumar et al., 2019). One of the effective ways to overcome these problems is to use nanotechnology. In nanotechnology, different organic and nonorganic nanofillers are used to improve the characteristics and performance of various films (Xiao et al., 2020).

ZnO has attracted much attention because it has a unique morphology and structure and significant and influential antibacterial and antifungal activity against a wide range of these microorganisms (Hassan Basri et al., 2020). Besides, ZnO has a friendly relation with the environment, easy preparation, and adaptability to the environment (Jamdagni et al., 2018). Food and Drug Administration (FDA) approved ZnO as a safe food additive. Zinc is an essential element for human health and its physiological activities, and each person needs 10 mg of zinc daily (Espitia et al., 2016). ZnO nanoparticles can be produced by different chemical and physical techniques (Vakulov et al., 2017).

Many studies have been conducted on the effect of ZnO nanoparticles on the properties of nanocomposite films and confirmed the improving effects of these nanoparticles on the functional and antimicrobial activity of the produced films (Babapour et al., 2021). The effect of nanoparticle size and morphology on the antibacterial and antifungal activities of ZnO nanoparticles also has been studied by researchers and shows the significant effect of the size and morphology of these nanoparticles on different microorganisms (Harun et al., 2019; Pariona et al., 2020). For example, Pariona et al. (2020) found that ZnO platelets showed higher antifungal activity than ZnO nanoparticles and nanorod shapes.

Although a few studies have been conducted on applying ZnO nanoparticles in different matrixes, to the best of our knowledge, the effect of ZnO‐N morphology on the functional and antimicrobial activity of biopolymer‐based films has not been studied. Therefore, this study aimed to investigate the effect of ZnO nanoparticle morphology on the different characteristics and antibacterial activity of tapioca starch nanocomposite films.

## 2 MATERIALS AND METHODS

### 2.1 Materials

Tapioca starch was purchased from SIM Company Bhd. ZnO‐ns and ZnO‐nr (40–100 nm) were received as gifts from the Physics school in Universiti Sains Malaysia. ZnO‐np and glycerol were purchased from Sigma Chemical Co. *Escherichia coli* O157:H7 cultures were supplied from the Organization of Scientific and Industrial Research of Iran. All other chemicals used were of analytical grade.

### 2.2 Preparation of nanocomposite films based on tapioca starch and ZnO nanoparticles

Different nano‐ZnO shapes were poured into the water at a concentration of 0.5%, 1.0%, and 2.0% w/w (based on the starch weight). The criteria for choosing this concentration range were based on Marvizadeh et al. (2017) with minor modification in preliminary experiments. The solutions then heated by a hot plate at 60°C for 1 hr. They are then placed on a shaker without heat for 24 hr to produce a homogeneous solution. The resulting solutions were homogenized in an ultrasonic bath at 40°C for 45 min. Based on the preliminary experiment to prepare tapioca starch films, 4% w/v tapioca starch was added to solutions containing various homogenized nano‐ZnO shapes. Preliminary experiments show that between common plasticizers (glycerol, sorbitol) at different concentrations (10%–60%), glycerol at 40% (w/w dried starch) was more consistent for tapioca film preparation. So at a constant 40% w/w (based on the dry matter), glycerol was used as a plasticizer. The starch dispersion containing the nanoparticles was heated to 85°C and kept at this temperature for 45 min until starch completely gelatinized while stirring on a hot plate. After complete gelatinization of the solution, it was cooled to 40°C. The control film was prepared similarly without the addition of nano‐ZnO. 90 g of the homogenized suspension was poured into casting plates with 16 × 16 cm^2^ to form a film. The films were dried under controlled conditions at 30°C and 50% relative humidity for 24 hr. The dried films were separated from the plates at 25°C and kept in a desiccator until further testing (Nafchi et al., 2013).

### 2.3 Characterization of bionanocomposite films

#### 2.3.1 Measurement of moisture content, solubility, and water absorption capacity

The moisture content of bionanocomposite films was measured using a thermogravimetric method described by Mohammadi Nafchi and Alias (2013). To measure the water solubility and water absorption capacity (WAC) of the films, methods described by Maizura et al. (2007) and Kiatkamjornwong et al. (2000) were used, respectively.

#### 2.3.2 Estimation of mechanical properties

The mechanical properties of bionanocomposite samples were measured according to ASTM standard method D882‐18 (ASTM, 2018). These properties include tensile strength, Young's modulus, and elongation at break. The film strips were cut to 100 mm × 20 mm and were conditioned for 48h (25°C and 55% RH). Measurements were performed using a texture analyzer (LLOYD, RS 232).

#### 2.3.3 Evaluation of water vapor permeability and oxygen permeability

The water vapor permeability (WVP) of film samples was evaluated according to ASTM Standard E96/E96M‐16 (ASTM, 2016). Briefly, the film was exposed to 100% relative humidity, and the degree of permeability was determined by weighing the cups every 1.5 hr for 48 hr at 25°C. The oxygen permeability (OP) of the films was evaluated according to ASTM standard method D3985‐17 (ASTM, 2017). Mocon Oxtran 2/21 system was used for this purpose.

#### 2.3.4 The UV–visible light transmission

The transmittance of UV and visible light for bionanocomposite films at the wavelength of 200–1,100 nm was recorded using a spectrophotometer. The transparent glass plate was used as a reference. In this evaluation, the wavelength of 200–400 nm was related to UV, 400–700 nm was related to visible light, and 700–1,100 was related to the near‐infrared wavelength (Akbariazam et al., 2016).

#### 2.3.5 Fourier transform infrared spectroscopy

Fourier transform infrared spectroscopy (FTIR) was used to investigate the chemical interaction. Samples of the film with a diameter of 1 cm and a thickness of 20 µm were prepared and squeezed between two KBr tablets. The tablet containing the film was placed inside the cell of FTIR spectrophotometer. The infrared spectrum was recorded in passing films in the range of 400–4000 cm^−1^ and with a resolution of 4 cm^−1^ (Zhou et al., 2006).

#### 2.3.6 Investigation of antibacterial activity by agar diffusion method (static method)

The film sample was cut into 5‐mm diameter disks and then placed on Mueller–Hinton agar plates. The plate had been previously seeded with 0.1 ml of inoculum containing approximately 10^5^–106 CFU/ml of *E*. *coli* bacteria. After that, the plate was incubated at 37°C for 24 hr. After incubation, the diameter of the inhibitory zone of the film disk was measured, and the inhibition zone area was calculated (Maizura et al., 2007).

### 2.4 Statistical analysis

All obtained data from tests were analyzed statistically using one‐way analysis of variance (ANOVA) and Duncan multirange test to identify the significant differences between samples at α = 0.05 using SPSS software version 22.0 (IBM Corp.).

## 3 RESULTS AND DISCUSSION

### 3.1 Effect of nano‐ZnO morphology on the moisture content of starch films

**FIGURE 1:**
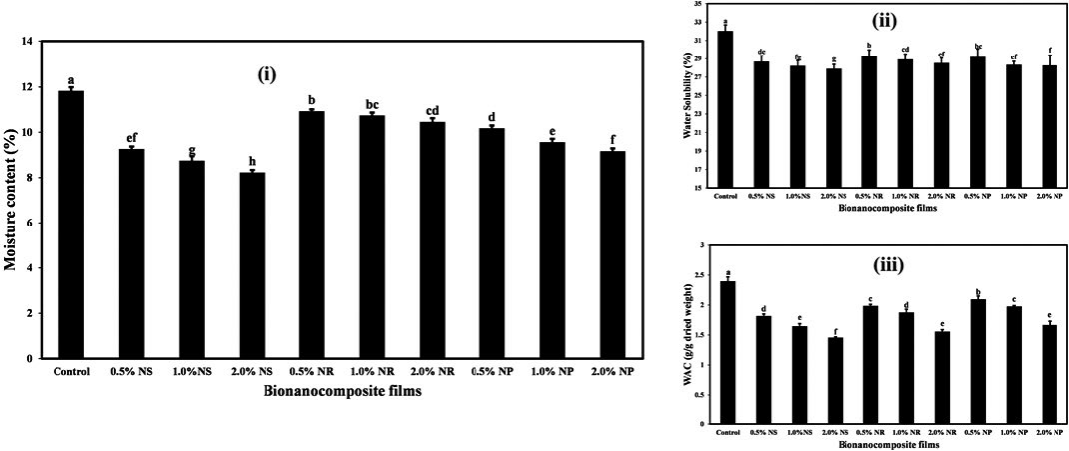
The moisture content (%) (i), water solubility (%) (ii), and water absorption capacity (g/g dried weight) (iii) of tapioca starch films containing different nano‐ZnO morphology. Bars represent mean (*n* = 5) ± *SD*. Different letters on the bars indicate a significant difference at 5% level of probability among starch films. NP, nanoparticle ZnO; NR, nanorod ZnO; NS, nanosphere ZnO

Moisture sensitivity in films is one of the problems that limit its use. The moisture content of the films containing different forms of ZnO nanoparticles at different concentrations is compared in Figure 1(i). As shown in the figure, incorporating different forms of ZnO nanoparticles and increasing their concentrations reduced the moisture content of the produced film compared to the control (*p* < .05). The highest reduction in the moisture content of the films was observed in the starch films containing the ZnO‐ns. The moisture content in control was 11.80%, and in the film containing 2%, the ZnO‐ns was reduced to 8.20%.

The lower moisture content of starch films containing nano‐ZnO to the control is due to the cohesive structure with high cohesion and less space and less hydrophilicity of ZnO nanoparticles than starch‐based polymer matrix (Alboofetileh et al., 2013). Moisture content reduction by ZnO‐ns is more than by other shapes. It is likely related to the comfortable placement of the nanosphere in the biopolymer structure. Nafchi et al. (2012) and Teymourpour et al. (2015) also reported a decrease in the moisture content of bionanocomposite films due to the incorporation of ZnO and TiO_2_ nanoparticles, respectively.

### 3.2 Effect of nano‐ZnO morphology on the water solubility of starch films

The packaging material must maintain the moisture levels in the packaged foods, so the moisture content, water solubility, and water absorption capacity of the films are critical parameters in the food packaging industry. The water solubility of starch films containing different forms of ZnO nanoparticles at different concentrations is given in Figure 1(ii). Results show that the highest water solubility was observed in control (31.99%), and incorporation of various forms of ZnO nanoparticles and increasing their concentrations in starch films significantly reduced the solubility of the nanocomposite films (*p* < .05). In films containing ZnO‐ns, ZnO‐nr, and ZnO‐np, the solubility decreased from 28.65%–27.87%, from 29.25%–28.50%, and from 29.17%–28.24%, respectively, by increasing nano‐ZnO concentration.

The reduction in solubility of biopolymer‐based films containing nano‐ZnO is probably related to the reactions between nano‐ZnO and starch in the film structure. Studies have shown that more hydrogen bonds are formed between nanoparticles and matrix compounds with increased nanoparticles in biofilms. As a result, free water molecules cannot react strongly with nanocomposite films than composite films alone (Tunç & Duman, 2010).

In studies conducted by Alebooyeh et al. (2012) and Nafchi et al. (2012), a reduction in the water solubility of biopolymer‐based films was observed due to the addition of ZnO nanoparticles and nanorods, respectively. These studies stated that incorporating nano‐ZnO resulted in the formation of more hydrogen bonds between the ZnO nanoparticles and the matrix, thus deactivating free water molecules and not interacting with nanocomposite films. Voon et al. (2012) also found that with increasing nanoclay and SiO_2_ nanoparticle levels in bovine gelatin‐based films, the film's solubility decreased significantly. They also attributed the decrease in film solubility to strong hydrogen bonds between the gelatin matrix and the nanoparticles. In a study conducted by Ngo et al. (2018), a decrease in the solubility of films based on pectin/alginate was observed due to the incorporation of nano‐ZnO.

### 3.3 Effect of nano‐ZnO morphology on water absorption capacity of starch films

Figure 1(iii) shows the water absorption capacity (WAC) amounts of the bionanocomposite films containing different nano‐ZnO. The control starch film had the highest WAC amount (2.39 g/g dried weight), and with the addition of different forms of nano‐ZnO and increasing their levels in films, the WAC amounts were significantly reduced (*p* < .05). The effect of ZnO‐ns on the reduction of the WAC was more remarkable than other forms. The lowest WAC was obtained in films containing 2% ZnO‐ns (1.45 g/g dried weight).

The reduction of WAC of starch‐based nanocomposite films due to the addition of different forms of nano‐ZnO is probably due to filling the empty spaces of the starch biopolymer matrix nanostructures. Nanostructures can react with hydroxyl groups of starch, limiting the site for the attachment of water to starch, which reduced the WAC of the films (Wu et al., 2009). Teymourpour et al. (2015) also observed that the addition of TiO_2_ nanoparticles to the film based on soluble soybean polysaccharide reduced the films' WAC. Mohammadi Nafchi et al. (2013) also reported a significant reduction in the WAC of film‐based bovine gelatin and sago starch due to the incorporation of ZnO‐nr. Alizadeh‐Sani et al. (2020) also achieved similar results when investigating the effect of ZnO nanoparticles on the WAC of sodium caseinate‐based films.

### 3.4 Effect of nano‐ZnO morphology on mechanical properties of starch films

The mechanical properties of films, including tensile strength (TS) and elongation (EB), are essential food packaging factors. TS indicates the maximum tensile force that film can withstand, and the EB indicates the maximum change in the length of the test sample before the break (Pereda et al., 2011). The mechanical properties of films are influenced by several factors such as the interactions between film compositions, temperature, and physical and chemical conditions (Sánchez‐González et al., 2011).

**FIGURE 2:**
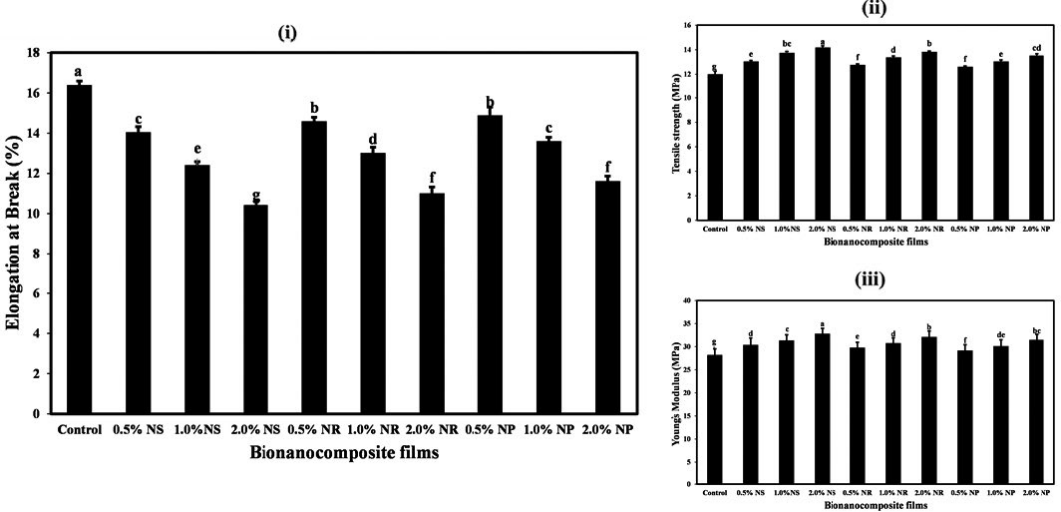
The elongation at break (%) (i), tensile strength (MPa) (ii), and Young's modulus (MPa) (iii) of tapioca starch films containing different nano‐ZnO morphology. Bars represent mean (*n* = 8) ± *SD*. Different letters on the bars indicate a significant difference at 5% level of probability among starch films. NP, nanoparticle ZnO; NR, nanorod ZnO; NS, nanosphere ZnO

The amounts of TS, Young's modulus (YM), and EB of the bionanocomposite films containing different forms of nano‐ZnOs are given in Figure 2. The results show that the lowest amounts of TS (11.97 MPa) and YM (28.13 MPa) and also the highest amount of EB (16.36%) were observed in control starch film. Increasing the concentration of different forms of nano‐ZnO in starch films led to a significant increase in TS and YM. It reduced the amount of EB of the nanocomposite films (*p* < .05). The effect of ZnO‐ns on changing the mechanical properties of starch films was significantly higher than other forms (*p* < .05). In general, the highest amounts of ST (14.15 MPa) and YM (32.74 MPa) and the lowest amount of EB (10.40%) were obtained in the films containing the highest level of ZnO‐ns (2%).

The first reason for the increase in tensile strength and a decrease in elongation at break of films due to nano‐ZnO is related to these films’ moisture content. Water acts as a plasticizer in the biocomposite network. Reducing the moisture content of films can reduce their flexibility and increase the strength and Yang's modulus of the resulting films (Müller et al., 2011). Increased tensile strength of nanocomposite films can be related to the uniform dispersion of nanoparticles in the matrix. The presence of nanoscale particles causes their unique surface properties and improves the strength of the polymers.

Huang et al. (2004) found that in bionanocomposites, due to mechanical and thermal operations on starch, hydrogen bonds between starch molecules are lost, and new bonds are formed between starch and nanoparticles. As a result, the mechanical properties of the produced films are improved. Teymourpour et al. (2015) also reported an increase in tensile strength and Young's modulus and a reduction in elongation at break of bionanocomposite films due to the increase in the level of TiO_2_ nanoparticles.

### 3.5 Effect of nano‐ZnO morphology on barrier properties of starch films

**FIGURE 3:**
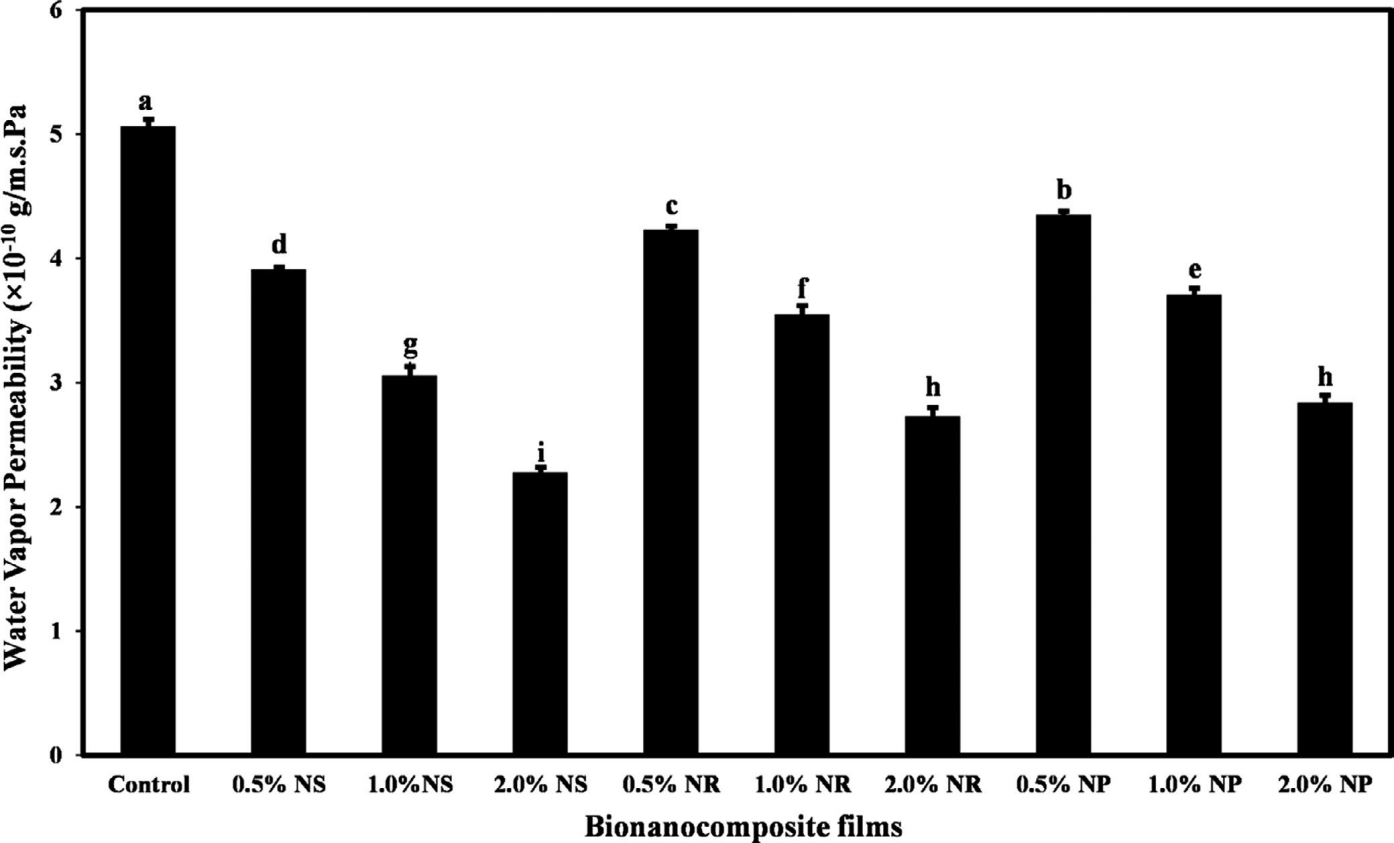
The water vapor permeability (g/m.s. Pa) of tapioca starch films containing different nano‐ZnO morphology. Bars represent mean (*n* = 3) ± *SD*. Different letters on the bars indicate a significant difference at 5% level of probability among starch films. NP, nanoparticle ZnO; NR, nanorod ZnO; NS, nanosphere ZnO

**FIGURE 4:**
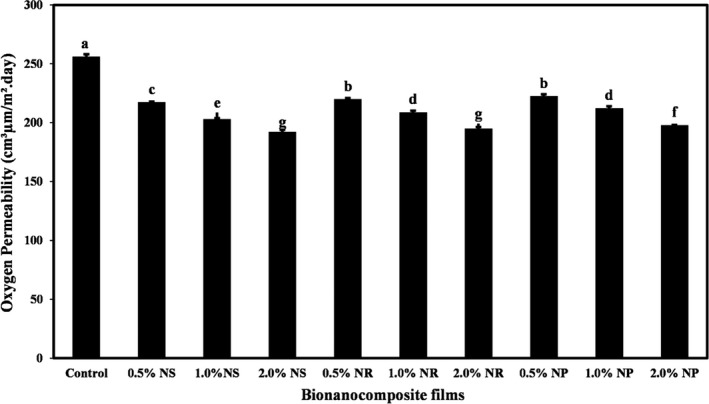
The OP (cm^3^ µm/(m^2^‐day)) of tapioca starch films containing different nano‐ZnO morphology. Bars represent mean (*n* = 3) ± *SD*. Different letters on the bars indicate a significant difference at 5% level of probability among starch films. NP, nanoparticle ZnO; NR, nanorod ZnO; NS, nanosphere ZnO

The WVP and OP amounts of nanocomposite films containing different forms of nano‐ZnO are shown in Figures 3 and 4. The control had the highest amounts of WVP (5.05 × 10^–10^ g/m.s. Pa) and OP (255.40 cm^3^ µm/(m^2^‐day)). Increasing the concentration of different forms of nano‐ZnO in starch films significantly decreased the WVP and OP (*p* < .05). ZnO‐ns had the greatest effect on decreasing WVP and OP of films. The lowest values of WVP (2.77 × 10^–10^ g/m.s. Pa) and OP (191.72 cm^3^ µm/(m^2^‐day)) were obtained in the films containing the highest level of ZnO‐ns.

The reduction in the WVP and OP of starch‐based nanocomposite films containing different forms of nano‐ZnO can be attributed to the fact that the presence of these nanoparticles in the biopolymer matrix complicates the path of molecules compared to starch polymer alone (Yu et al., 2009). Since the main disadvantage of films based on biopolymers is the high speed of water vapor passage, the use of bionanocomposite in comparison with biopackaging can reduce the relative moisture transfer rate (Thellen et al., 2005). In general, the improvement of the barrier properties of bionanocomposite films is due to the formation of a coherent and sense structure (Han et al., 2018).

Ng et al. (2018), Teymourpour et al. (2015), and Nafchi et al. (2012) have reported a significant reduction in the WVP and OP of films based on biopolymers due to the incorporation of nanoparticles. Alizadeh‐Sani et al. (2020) and Mirjalili and Yasini Ardekani (2017) also demonstrated that the addition of nano‐ZnO to starch film formulation and sodium caseinate‐based film led to a decrease in the WVP of bionanocomposite films, respectively. Zeppa et al. (2009) and Marvizadeh et al. (2017) observed a decrease in OP of starch‐based films and bovine gelatin–tapioca starch films due to the addition of nanoclay and ZnO nanorods, respectively.

### 3.6 Effect of nano‐ZnO morphology on the UV–visible light transmission of starch films

**FIGURE 5:**
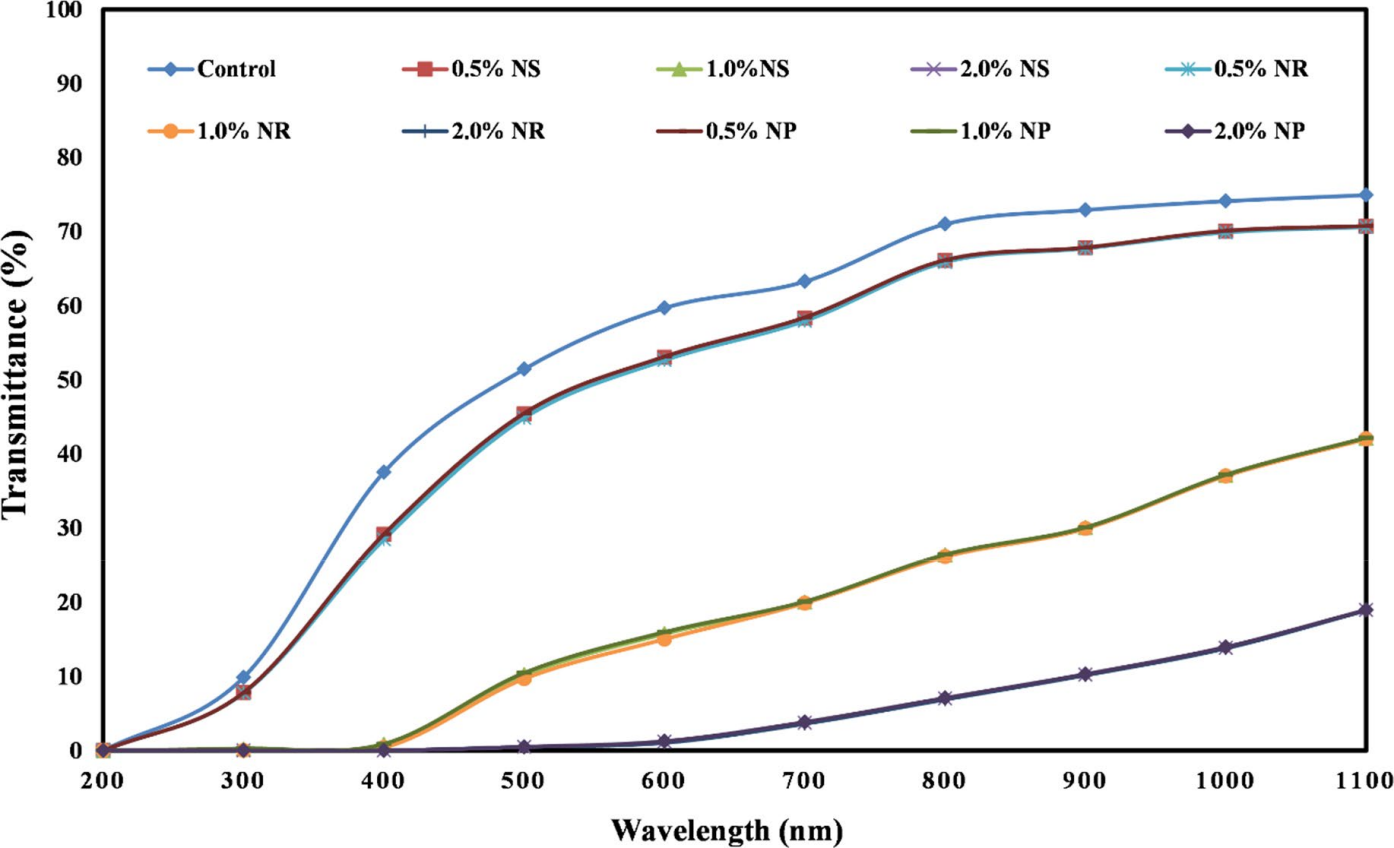
The UV–visible transmittance (%) of tapioca starch films containing different nano‐ZnO morphology. NP, nanoparticle ZnO; NR, nanorod ZnO; NS, nanosphere ZnO

In Figure 5, the percentage of UV light passing through starch‐based nanocomposite films containing different forms of ZnO nanoparticles is shown. As expected, with increasing the concentration of different forms of nano‐ZnO in the film samples, the percentage of UV light transmission decreased significantly. The highest amount of UV light transmission was related to the control sample, and the lowest amount was observed in films containing the highest levels of nano‐ZnO (2.0%). UV rays do not pass through nanocomposite biofilms due to the intense absorption or scattering of UV light by nano‐ZnO.

Nafchi et al. (2014) showed that by applying ZnO nanoparticles in starch‐based nanocomposite films, nanoparticles could limit the passage of UV light severely. Rouhi et al. (2013) also observed a reduction in the percentage of UV light passing through fish gelatin‐based films due to the addition of ZnO nanorods. Similar results were reported in a study by Ngo et al. (2018). These researchers showed that edible films based on pectin–alginate containing ZnO nanoparticles had a lower UV light transmission percentage than the control film. Shahabi‐Ghahfarrokhi et al. (2015) also obtained similar results when they studied the effect of biopolymer films containing ZnO nanoparticles on the percentage of UV light transmission.

### 3.7 Effect of nano‐ZnO morphology on the FTIR spectra of starch films

**FIGURE 6:**
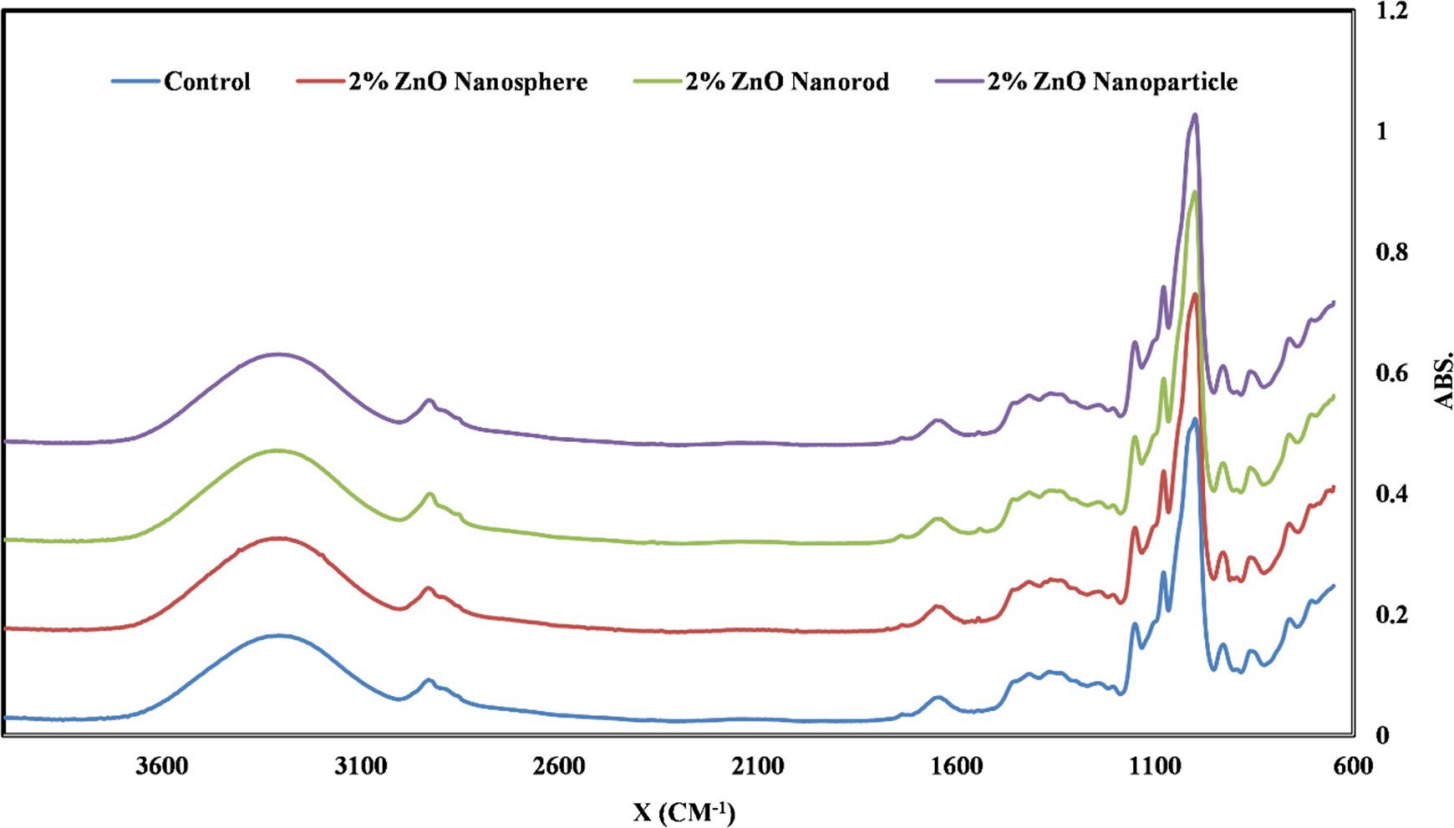
The FTIR spectrum of tapioca starch films containing different morphology of nano‐ZnO. FTIR, Fourier transform infrared spectroscopy; NP, nanoparticle ZnO; NR, nanorod ZnO; NS, nanosphere ZnO

The different functional groups and bonds in the chemical structure of materials absorb certain infrared (IR) radiation frequencies. FTIR spectroscopy is a suitable tool in the study of structural changes in composites. Figure 6 shows the FTIR spectrum of starch‐based nanocomposite films containing different forms of ZnO‐N at the level of 2.0%. The specific absorption peak of pure starch is in the wavenumber of 3,000 cm^−1^. This is due to the bond between the H and OH groups, which causes complex tensile vibrations related to the free intermolecular and intramolecular hydroxyl groups present in the natural biopolymer structure of starch (Ibrahim, 2011). The wavenumber of 1,152 cm^−1^ and the wavenumbers of 1,015 and 1,052 cm^−1^ are related to the bond between C‐O of the C‐O‐H group and the C‐O bond of the C‐O‐C group in the anhydroglucose rings, respectively (Rosa et al., 2010). Absorption bands 847 and 966 cm^−1^ are related to the vibration of C‐O‐C ring of starch. Peaks observed in the range of 1,306–1,570 cm^−1^ are attributed to C‐H bonds and O‐H alcohol group bonds (Božanić et al., 2009). It is quite clear that no new functional groups have emerged after the incorporation of ZnO nanoparticles. Thus, the interaction between the film polymer and the ZnO nanoparticles is only physical.

Nafchi et al. (2012) added ZnO‐nr to the sago starch films. They did not observe any new functional groups in the films and concluded that ZnO‐nr created a physical interaction with the film matrix. Nafchi et al. (2013) also added ZnO‐nr to the gelatin‐based films and did not report any new functional groups in the resulting films. Alizadeh‐Sani et al. (2020) also observed that incorporating ZnO‐np into sodium caseinate films did not show significant changes in the molecular structure of the polymer matrix, and no new bond was formed.

### 3.8 Effect of nano‐ZnO morphology on the antibacterial activity of starch films against *Escherichia coli*

*Escherichia coli* is the most important foodborne pathogenic. It is transmitted through oral route or fecal, and it should, under no circumstances, be present in any food and food products. So, *E*. *coli* count considered as an indicator bacterium in food safety and hygiene (Ekici & Dümen, 2019).

**FIGURE 7:**
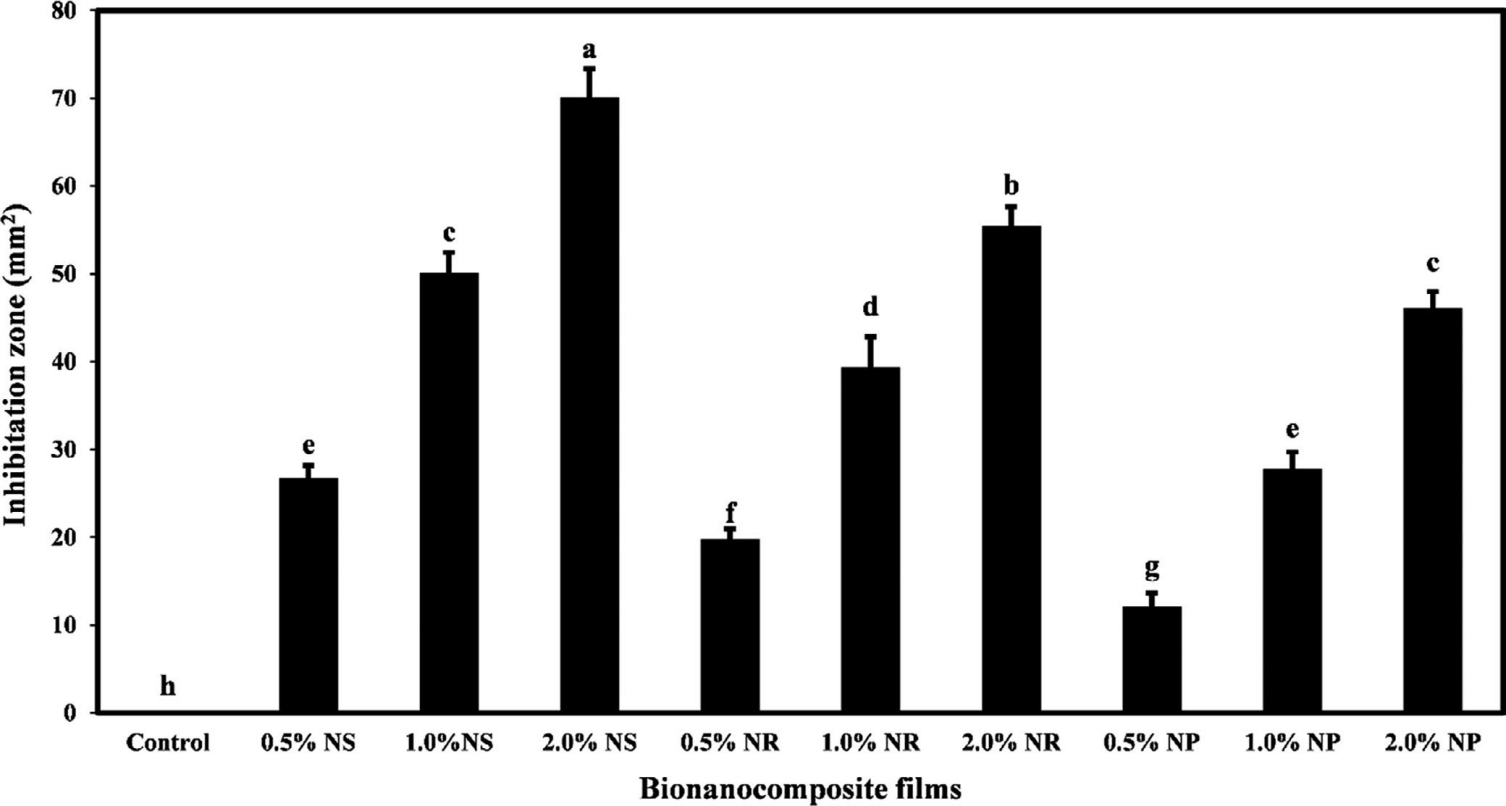
The *Escherichia coli* growth inhabitation zone (mm^2^) of tapioca starch films containing different nano‐ZnO morphology. Bars represent mean (*n* = 3) ± *SD*. Different letters on the bars indicate a significant difference at 5% level of probability among starch films. NP, nanoparticle ZnO; NR, nanorod ZnO; NS, nanosphere ZnO

Figure 7 compared the antibacterial activity of bionanocomposite films based on tapioca starch‐containing different nano‐ZnO morphology against *E*. *coli* (Gram‐negative) bacteria. It shows that the control film had no antibacterial activity against this bacterium. The antibacterial activity was increased by adding different nano‐ZnO forms to biocomposite films and increasing their concentrations. The area of the growth inhabitation zone against *E*. *coli* increased significantly (*p* < .05). The antibacterial activity of ZnO‐ns was higher than ZnO‐nr and ZnO‐np. The highest area of the growth inhabitation zone was related to the level of 2.0% ZnO‐ns.

In general, various mechanisms have been proposed for the antimicrobial action of metal nanoparticles, the most important of which are the catalytic oxidation and reduction activity that affects the DNA, the active site of enzymes, and the activity of ribosomes. These functions disrupt the metabolic activity of bacteria (Bruna et al., 2012; Llorens et al., 2012). Induction of reactive oxygen species (ROS) such as hydrogen peroxide, hydroxyl radicals, and superoxide (Emamifar et al., 2010) and cell wall damage are also some of the proposed mechanisms for the antimicrobial activity of metal nanoparticles (Li et al., 2011). When nano‐ZnO is placed in the vicinity of bacteria cells, the reaction between the positive charge of ZnO ions and the negative charge of bacteria cell occurs. As a result, the electrostatic force is generated and destroys the bacteria cell membrane and leaks intracellular materials (Ramalingam et al., 2016).

The researchers found that the smaller the nanoparticle size, the larger the surface area and the more effective it was at killing bacteria (Agnihotri et al., 2014; Torres et al., 2013). In general, the binding of the active agent to the bacterium depends on the surface area. Therefore, as the surface area of the active agent increases, more reactions occur (Morones et al., 2005). Hence, the shape of nanomaterials affects their antibacterial activity.

Raza et al. (2016) also observed that the antibacterial activity of silver nanoparticles depends on the shape and size of particles. The highest antibacterial activity against *Pseudomonas aeruginosa* and *E*. *coli* was observed in spherical nanoparticles with the smallest particle size, and nanoparticles with a triangular shape were second. Harun et al. (2019), in the study of the effect of the shape of nano‐ZnO on the antibacterial activity of these nanoparticles, showed that ZnO nanoflakes and nanospheres had the highest antibacterial activity against *E*. *coli*. Other researchers have suggested that nano‐ZnO with a smaller size and higher surface area can show better antimicrobial activity (da Silva et al., 2019).

## 4 CONCLUSION

In this study, the morphological effect of nano‐ZnO on the physical, chemical, mechanical, and antibacterial properties of tapioca starch films was investigated. The results demonstrated that incorporating different forms of nano‐ZnO and increasing their concentration in nanocomposite films improved the physical, chemical, and mechanical properties and strength of the produced films. The resulting nanocomposite starch films had good barrier properties against UV light and showed significant antibacterial activity against *E*. *coli*. Among different shapes of nano‐ZnO, the highest effect on the functional and antibacterial properties of starch‐based films was nanosphere forms of ZnO. Generally, this study showed that the shape of ZnO nanoparticles has a significant effect on their performance.

## CONFLICT OF INTEREST

The authors declare no conflict of interest.

## AUTHOR CONTRIBUTIONS

**Naser Tamimi:** Data curation (equal); Formal analysis (equal); Investigation (lead); Resources (equal); Writing‐original draft (equal). **Abdorreza Mohammadi Nafchi:** Conceptualization (lead); Data curation (equal); Project administration (equal); Supervision (equal); Validation (equal). **Hamid Hashemi‐Moghaddam:** Supervision (equal); Validation (equal); Writing‐review & editing (equal). **Homa Baghaei:** Methodology (equal); Supervision (equal); Validation (equal); Writing‐review & editing (equal).

## ETHICAL APPROVAL

This study does not involve any human or animal testing.

## Data Availability

The data that support the findings of this study are available from the corresponding author, upon reasonable request.
